# Excavatum & Exercise: Digging for causes of dyspnoea

**DOI:** 10.1186/1749-8090-10-S1-A276

**Published:** 2015-12-16

**Authors:** K Mazhar, I Cliff, N Watson, CMR Satur

**Affiliations:** 1 The Heart Centre, Royal Stoke University Hospital, Stoke-on-Trent, Staffordshire, ST46QG, UK

## Background/Introduction

Pectus Excavatum is the most common congenital chest wall deformity (90%) and the documented incidence ranges between 0.1% - 0.3%. Patients with Pectus Excavatum can suffer with body image and psychological issues. Indications for surgical correction remains controversial although a decrease in exercise tolerance is frequently overlooked. A general consensus persists that Pectus Excavatum is a cosmetic defect with no physiological consequences.

## Aims/Objectives

In this study we investigated the functional exercise capacity of patients with Pectus Excavatum (PE).

## Method

Between Feb 2006 and March 2015: 44 patients presented to our institution with symptomatic PE. 29 (26 male : 3 female) patients underwent complete investigational study including Computed Tomography (CT) of the thorax Cardiopulmonary Exercise tolerance (CPEX) testing including measurement of Cardiovascular parameters: Maximal oxygen consumption (VO2 max) ; normal > 85% predicted. Anaerobic Threshold (AT)=: change from aerobic to anaerobic metabolism (normal range 47 -64%). O2 pulse (an indirect measure of stroke volume; normal > 90% predicted). Respiratory parameters: Ventilatory reserve (VE BTPS) ; normal > 85% predicted. Peak exercise. End Tidal CO2 (PETCO2) ; normal 30 - 38 mmHg.

## Results

Results Mean Age 22.4 years (13 - 33 years). All presented with symptoms of dyspnoea /fatigue /dysphagia. Mean VO2 max was 78% (51 - 102%). VO2 max was sub-normal in 19 (65%) indicating cardiac dysfunction. 15 (51%) patients had reduced AT. 15 patients had reduced VO2 /HR. 28 (96.5%) had marked increase/unused ventilatory reserve. 14 (48%) had a marked increase in PETCO2, inability to expire CO2.

## Discussion/Conclusion

Pectus Excavatum is associated with significant compromise in cardiopulmonary physiology (65%).The aetiology of the defect is mainly cardiac or respiratory in nature with some having a mixed picture. We advocate the routine use CPEX testing in PE patients prior to corrective surgery. These results support the premise that most patients with severe anatomical PE defect also have an associated severe physiological defect.

